# The Role of High-Level Processes for Oscillatory Phase Entrainment to Speech Sound

**DOI:** 10.3389/fnhum.2015.00651

**Published:** 2015-12-02

**Authors:** Benedikt Zoefel, Rufin VanRullen

**Affiliations:** ^1^Université Paul SabatierToulouse, France; ^2^Centre de Recherche Cerveau et Cognition (CerCo), CNRS, UMR5549, Pavillon Baudot CHU PurpanToulouse, France

**Keywords:** EEG, oscillation, phase, entrainment, high-level, speech, auditory, intelligibility

## Abstract

Constantly bombarded with input, the brain has the need to filter out relevant information while ignoring the irrelevant rest. A powerful tool may be represented by neural oscillations which entrain their high-excitability phase to important input while their low-excitability phase attenuates irrelevant information. Indeed, the alignment between brain oscillations and speech improves intelligibility and helps dissociating speakers during a “cocktail party”. Although well-investigated, the contribution of low- and high-level processes to phase entrainment to speech sound has only recently begun to be understood. Here, we review those findings, and concentrate on three main results: (1) Phase entrainment to speech sound is modulated by attention or predictions, likely supported by top-down signals and indicating higher-level processes involved in the brain’s adjustment to speech. (2) As phase entrainment to speech can be observed without systematic fluctuations in sound amplitude or spectral content, it does not only reflect a passive steady-state “ringing” of the cochlea, but entails a higher-level process. (3) The role of intelligibility for phase entrainment is debated. Recent results suggest that intelligibility modulates the behavioral consequences of entrainment, rather than directly affecting the strength of entrainment in auditory regions. We conclude that phase entrainment to speech reflects a sophisticated mechanism: several high-level processes interact to optimally align neural oscillations with predicted events of high relevance, even when they are hidden in a continuous stream of background noise.

## Phase Entrainment as a Tool for Input Gating

**Figure 1:**
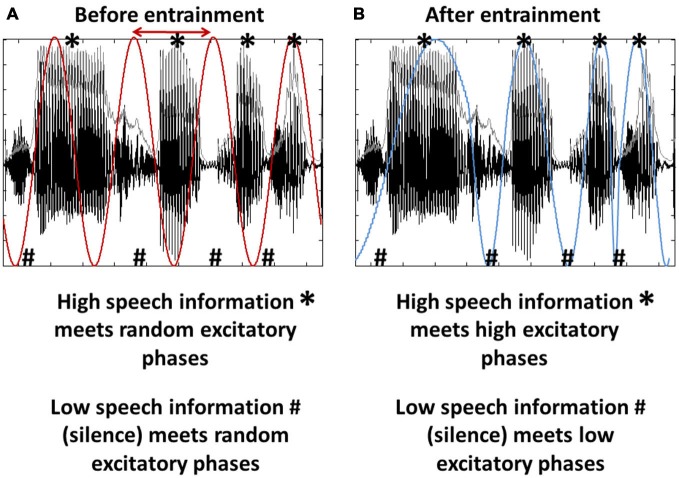
**Entrainment as a tool for input gating. (A)** Brain oscillations (red) are unrelated to the stimulus input, here a segment of speech sound. Note that both oscillation and speech sound are rhythmic (~4 Hz) and that the speech input consists of phases of high (*) and low (#) informational content. Both phase and frequency (the latter to a certain extent; Ghitza, 2013, 2014) of the oscillations can be adjusted to match the input rhythm (red arrow), a phenomenon called phase entrainment. **(B)** Phase entrainment results in an alignment of the oscillation’s high and low excitability phases (blue) with the input’s high and low informational content. It can thus be used as a tool for input gating.

In virtually every situation of our life, the brain has to cope with an enormous amount of incoming information, only a fraction of which is essential for the scene’s interpretation or resulting behavior. Clearly, the brain must have evolved strategies to deal with this vast influx, and both amplification of relevant input and suppression of irrelevant information will be critical for survival. Based on recent research, one prominent tool for the described purpose are neural oscillations, assumed to reflect cyclic changes in the excitability of groups of neurons (Buzsáki and Draguhn, 2004; Rajkai et al., 2008; Mazzoni et al., 2010). These *endogenous* fluctuations in neural excitability *per se* might seem without function at first glance, as long as they are *passive* and unrelated to the environment (Figure 1A). However, as previous studies showed, both on a theoretical (Schroeder et al., 2008, 2010; Schroeder and Lakatos, 2009; Ghitza, 2011; Morillon et al., 2015) and experimental level (Lakatos et al., 2005, 2008, 2013; Stefanics et al., 2010; Besle et al., 2011; Henry and Obleser, 2012; Henry et al., 2014; Morillon et al., 2014; Nozaradan, 2014; O’Connell et al., 2014; Arnal et al., 2015; Park et al., 2015), these oscillations might become an interesting tool when introducing the possibility that they can be *controlled* by the brain. By using the low and high excitability phases of those oscillations, the brain might *actively* “decide” what part of the incoming information should be amplified (the information coinciding with the oscillation’s high excitability phase) and what part should be suppressed (the information coinciding with the oscillation’s low excitability phase; Figure 1B). This phenomenon, the synchronization of an oscillatory system (here: brain oscillations) with external input has been termed *phase entrainment* (Schroeder and Lakatos, 2009). Of course, this kind of “input gating” can only be exploited functionally if the input is (1) rhythmic (i.e., predictable), (2) has a relatively stable frequency that the brain can entrain to, and (3) alternates between low and high informational content. Interestingly, one of the most salient stimuli in everyday life fulfills these criteria: speech sound. Although only considered “pseudo-rhythmic” (Cummins, 2012; but see Ghitza, 2013), the frequency of the speech envelope (roughly defined as the sum of energy across sound frequencies at a given point in time; shown as gray line in Figure 1) is relatively stable between 2 and 8 Hz and phases of low phonetic information (e.g., the silence between syllables) rhythmically alternate with phases of high phonetic information.

Indeed, the number of studies reporting an adaptation of neural oscillations to the envelope of speech sound is increasing continuously (Ding and Simon, 2012a,b, 2013, 2014; Peelle and Davis, 2012; Zion Golumbic et al., 2012, 2013b; Ding et al., 2013; Gross et al., 2013; Horton et al., 2013; Peelle et al., 2013; Power et al., 2013; Steinschneider et al., 2013; Doelling et al., 2014; Millman et al., 2015; Park et al., 2015). But not only speech sound is able to evoke an entrainment of neural oscillations, even simple stimuli, such as pure tones, have been found to produce phase entrainment (Stefanics et al., 2010; Besle et al., 2011; Gomez-Ramirez et al., 2011; Zoefel and Heil, 2013). Furthermore, rhythmic fluctuations in stimulus amplitude (which are present in both trains of pure tones and speech sound) introduce fluctuations at a level of auditory processing as low as the cochlea, a notion that is obviously not compatible with phase entrainment as an active or “high-level” process. Similar concerns have been raised by several authors in the last years (Obleser et al., 2012; Zion Golumbic et al., 2012; Ding et al., 2013; Peelle et al., 2013; Zoefel and Heil, 2013; Ding and Simon, 2014; VanRullen et al., 2014). Based on these concerns, it might be argued that a mere “following” of stimulus amplitude (leading to a series of evoked potentials) and the entrainment of endogenous neural oscillations might be completely different processes with different types of underlying mechanisms. Most studies investigating phase entrainment did not differentiate these components and might have measured a mix of evoked and entrained responses. For the sake of simplicity, and because it is not straightforward to disentangle the two, we will call both processes “phase entrainment” throughout this manuscript, to describe an experimentally observable metric without assuming one or the other underlying process. However, we dedicated the last paragraph of Section “Phase Entrainment to High-Level Features of Speech Sound” to this issue, in which the controversy “evoked vs. entrained” is discussed in more detail.

The issues outlined in the previous paragraph lead to the fact that the role of high-level processes for phase entrainment to speech sound is far from clear. Nevertheless, significant progress has been made within the last decade, and the aim of this review is to summarize the obtained results in a systematic way. The scope of this review is not a summary of existing literature showing an alignment between brain oscillations and speech sound, as comprehensive reviews have been published recently (Peelle and Davis, 2012; Zion Golumbic et al., 2012; Ding and Simon, 2014). Rather, we will focus on high-level processes that can modulate or even underlie this alignment. Critically, it is necessary to differentiate between (i) high-level *modulations* of phase entrainment and (ii) high-level *entrainment*: In (i), phase entrainment can be produced as a “following” response to a low-level rhythmic stimulus sequence (potentially in early brain areas, as early as the cochlea); however, the entrainment is modulated by high-level processes that include attention or predictions. In this review, low-level features of speech are defined as stimulus amplitude and spectral content, as those two properties can passively entrain the lowest level of auditory processing and evoke steady-state-potential-like (ASSR; Galambos et al., 1981) fluctuations in the cochlea. In contrast to (i), high-level *entrainment* (ii) represents phase entrainment that can be observed even in the absence of systematic fluctuations of low-level properties. In this case, a simple “following” of stimulus amplitude is not possible anymore. Thus, it is the process of phase entrainment *itself* that operates on a higher level, as a certain level of processing is required in order to adjust to the rhythm of high-level features. Convincing results have been obtained in the last years for both types of high-level processes, and we will address them in separate sections. We conclude this review with a section dedicated to the role of intelligibility for phase entrainment to speech sound, as the influence of semantic information on the brain’s adjustment to speech is currently a strongly debated topic.

## High-Level Modulations of Phase Entrainment to Speech Sound

Certain cognitive processes, such as attention, expectation or interpretation, are often considered “high-level” functions of the human brain, as they require, for instance, evaluation, selection, and the comparison of the actual stimulation with experience (Lamme and Spekreijse, 2000; Gilbert and Li, 2013; Peelen and Kastner, 2014). A modulation of phase entrainment to speech sound by those cognitive processes would argue for phase entrainment being a process that is not restricted to a purely sensory mechanism, but rather the active gating mechanism (or “active sensing”; Schroeder et al., 2010) that was explained above. Indeed, there is accumulating evidence for phase entrainment critically relying on attentional processes: one example is based on the so-called “cocktail party effect” (Cherry, 1953), describing a situation of several competing speakers, one of which has to be selected within the “noise” of the other, potentially distracting, speakers.

**Figure 2:**
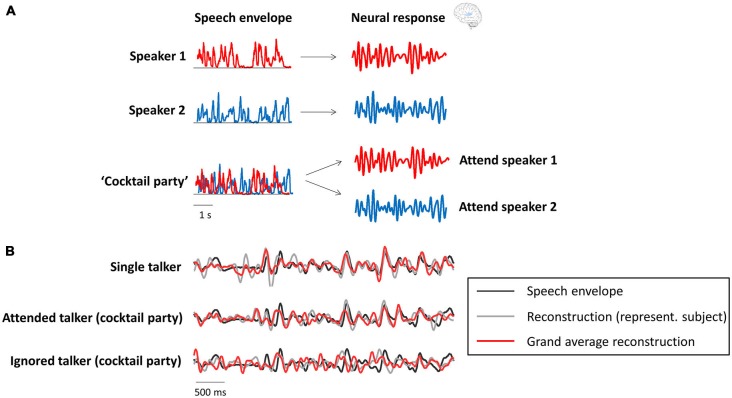
**Neural oscillations as a tool for attentional selection during a “cocktail party”. (A)** Theoretical background (modified with permission from Zion Golumbic et al., 2012, copyright Elsevier). Recorded neural activity in the delta/theta band (right column) aligns with the speech envelope (left column) of the respective speaker (red and blue), when presented separately. In a multi-speaker scenario (“cocktail party”), the recorded data will reflect the attended, but not necessarily (or to a smaller degree) the unattended speech envelope. **(B)** Actual data (modified with permission from Zion Golumbic et al., 2013b, copyright Elsevier) confirms the theoretical background. The speech envelope reconstructed from the recorded data (gray: single subject; red: averaged across subjects) strongly resembles the speech envelope (black) of the attended, but not the unattended speaker.

Several recent studies have shown a relation between the “cocktail party effect” and phase entrainment (the theoretical background is shown in Figure 2A and underlined by experimental results in Figure 2B). In Kerlin et al. (2010), two different speech streams were presented to the participants, one to each ear, and they were asked to selectively attend one of those two competing streams. They found that the representation of the attended speech stream in the delta/theta range (~2–8 Hz; the dominant frequency range of the speech envelope) of the electroencephalogram (EEG) signal was enhanced compared to that of the unattended stream. In other words, phase-locking between the EEG signal and the speech envelope of the attended stream was stronger than that between the EEG signal and the unattended stream. A similar paradigm was used in the studies by Ding and Simon (2012a), Horton et al. (2013) and Zion Golumbic et al. (2013b) in magnetoencephalographic (MEG), EEG and intracranial recordings in human subjects, respectively. All studies confirmed the finding that the phase of delta/theta brain oscillations “tracks” the envelope of speech sound, and that this “tracking” is enhanced when the speech is attended in a multi-speaker scenario. Interestingly, all studies reported that even the unattended speech signal is still represented (albeit weakly) in lower-level auditory cortices (i.e., regions closely related to sensory processing). However, as shown in the work by Zion Golumbic et al. (2013b), this unattended signal is “lost” in higher-level (e.g., frontal) regions. Ding and Simon (2012a) demonstrated that only the representation of the attended (and not the unattended) speech envelope varies as a function of stimulus intensity. This finding is important, because it suggests that attended and unattended inputs are processed separately in the brain, and that the alignment between neural phase and speech rhythm is used to form individual “auditory objects” (for a review on this notion, see Simon, 2015). In line with the notion of phase entrainment as an “amplifier-attenuator mechanism” (see “Phase Entrainment as a Tool for Input Gating”), Horton et al. (2013) reported cross-correlations between speech envelope and EEG signal for both attended and unattended streams, but with opposite signs, suggesting that phase entrainment is indeed used to amplify one stream while the other is attenuated. Finally, it has been shown in several studies that the speech envelope can be reconstructed (i.e., it can be identified which stimulus the listener is attending) in multi-speaker (Ding and Simon, 2012a; Zion Golumbic et al., 2013b; O’Sullivan et al., 2015) or noisy environments (Ding and Simon, 2013) by using the delta/theta phase of neural oscillations (but also their gamma power; Mesgarani and Chang, 2012; Zion Golumbic et al., 2013b). It is possible that in those kind of situations, where one speech stream has to be actively extracted from a noisy environment, attention is of particular importance for phase entrainment to speech sound, whereas clear speech can be processed largely independently of attention (Wild et al., 2012).

Not only attention can be considered a high-level process: predictions reflect a comparison between present and previous experiences and its projection to the future and must therefore involve high-level functions of the brain (Friston, 2005; Arnal and Giraud, 2012). Indeed, it has been shown that predictions do influence phase entrainment to speech sound. For instance, in the “cocktail party” scenario described above, Zion Golumbic et al. (2013a) paired the auditory speech input with the speaker’s face and found that phase entrainment to the speech envelope was significantly enhanced by this visual input. Similar results were obtained by Arnal et al. (2011) using congruent and incongruent audiovisual stimuli (syllables) and by Luo et al. (2010) when subjects were watching audiovisual movies. A common interpretation of these findings is that, due to the slight delay between visual and auditory components of a conversation (the visual input preceding the auditory one), the former can be used to predict the timing of speech sound, thus enabling a better alignment between the oscillatory phase and speech envelope (Arnal et al., 2009, 2011; Zion Golumbic et al., 2013a; Perrodin et al., 2015; for a review, summarizing several existing theories, see Peelle and Sommers, 2015). A phase-reset of neural oscillations in primary auditory cortex by visual input seems to be an important underlying mechanism (Thorne and Debener, 2014; Mercier et al., 2015; Perrodin et al., 2015). Although this would indicate an involvement of low or intermediate hierarchical levels, we emphasize here that a purely low-level mechanism is insufficient to explain many findings reported in the literature. For instance, introducing an additional delay between visual and auditory input disrupts the benefits of additional visual information for speech processing and incongruent visual information (which would result in a similar phase-reset as congruent information, assuming a purely low-level process) does not result in enhanced phase entrainment (e.g., Crosse et al., 2015; for a review, see Peelle and Sommers, 2015) but instead generates an increased neural response associated with error processing (Arnal et al., 2011). Finally, using a McGurk paradigm (McGurk and MacDonald, 1976; van Wassenhove et al., 2005) were able to show a correlation between the amount of prediction conveyed by the preceding visual input for the upcoming speech and the latency of speech processing. Together, these results speak for a mechanism that is tailored to speech-specific processing (Crosse et al., 2015) and against a purely low-level mechanism. The timing of the cross-modal phase-reset seems to have evolved in such a way that oscillations in the auditory system arrive at their high excitability phase exactly when the relevant auditory input is expected to be processed (Lakatos et al., 2009; Thorne and Debener, 2014). Finally, recent research suggests that not only the visual, but also the motor system plays a critical role for an efficient adjustment of excitability fluctuations in auditory cortex to expected upcoming events (Fujioka et al., 2012; Doelling et al., 2014; Morillon and Schroeder, 2015; Morillon et al., 2015). For instance, it has been suggested that the motor system possesses its own representation of expected auditory events and can therefore prepare oscillations in auditory cortex for relevant upcoming stimuli (Arnal and Giraud, 2012; Arnal, 2012). This mechanism might underlie recent findings describing an enhanced segregation of relevant and irrelevant auditory events in the presence of rhythmic tapping (Morillon et al., 2014).

Not an experimental, but rather an analytical proof of high-level processes involved in phase entrainment was provided by two recent studies (Fontolan et al., 2014; Park et al., 2015). Fontolan et al. (2014) used Granger causality (Granger, 1969), applied on data recorded intracranially in human subjects, to demonstrate that information reflected in the phase of low-frequency oscillations in response to speech sound travels in top-down direction from higher-order auditory to primary auditory regions, where it modulates the power of (gamma) oscillations at higher frequencies. Park et al. (2015) analyzed their data, recorded with MEG, using transfer entropy measures (Schreiber, 2000). They were able to show that frontal and motor areas can modulate the phase of delta/theta oscillations in auditory cortex (note that the spatial resolution in this study was lower than for intracranial recordings. It is thus unclear whether these delta/theta oscillations correspond to those in higher-order auditory or primary auditory cortices described in Fontolan et al., 2014). Importantly, these top-down signals were correlated with an enhanced phase entrainment to speech sound when tracking of forward vs. backward speech was compared, indicating that higher-level processes can directly control the alignment between neural oscillations and speech sound.

The results described in this section strongly support the view that phase entrainment is a tool for attentional selection (Schroeder and Lakatos, 2009), filtering out irrelevant input and enhancing the representation of the attended stimulus in the brain. Predictions, potentially reflected by top-down mechanisms, help “designing” this filter by providing the timing for the alignment of “good” and “bad” phases of the oscillation to predicted relevant and irrelevant stimuli, respectively. This mechanism would not only help selecting relevant input in a noisy background, but also parse the speech signal at the same time: here, one cycle of the aligned oscillation would represent one segment of information (or “chunk”; Ghitza, 2011, 2013, 2014; Doelling et al., 2014) that is analyzed by means of faster oscillations (Giraud and Poeppel, 2012; Luo and Poeppel, 2012; for reviews, see Peelle and Davis, 2012; Ding and Simon, 2014). Thus, phase entrainment could function as a means of discretization (equivalent ideas are mentioned by Peelle and Davis, 2012; Zion Golumbic et al., 2012), similar to “perceptual cycles” commonly observed in vision (VanRullen et al., 2014).

## Phase Entrainment to High-Level Features of Speech Sound

**Figure 3:**
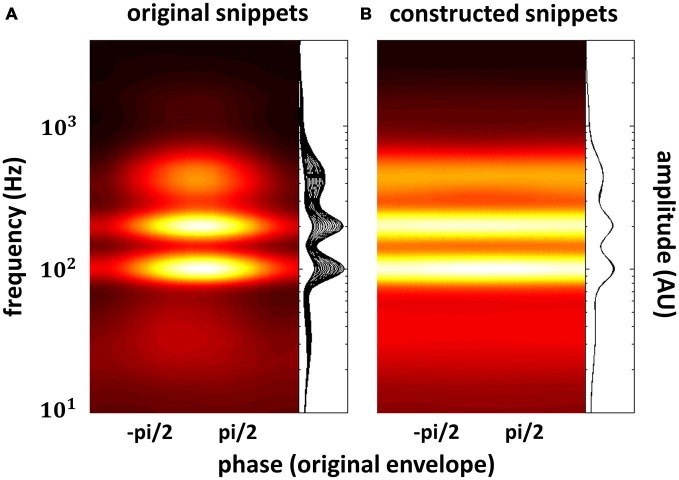
**Everyday speech sound (A) contains pronounced fluctuations in spectral energy (color-coded or shown as a family of curves; one curve for each phase bin of the speech envelope) that depend on the phase of the speech envelope.** These (low-level) rhythmic fluctuations in energy *per se* might result in an apparent alignment between neural activity and speech envelope, as strong neural excitation (here at phase 0, due to high spectral energy) periodically alternates with weak neural excitation (here at phase ± pi, due to low spectral energy). Genuine high-level phase entrainment requires stimuli without those systematic fluctuations in spectral energy, as shown in **(B)**. The construction of those stimuli has recently been reported (Zoefel and VanRullen, 2015b), and results obtained there are described in this review. Reproduced with permission from Zoefel and VanRullen (2015b).

**Figure 4:**
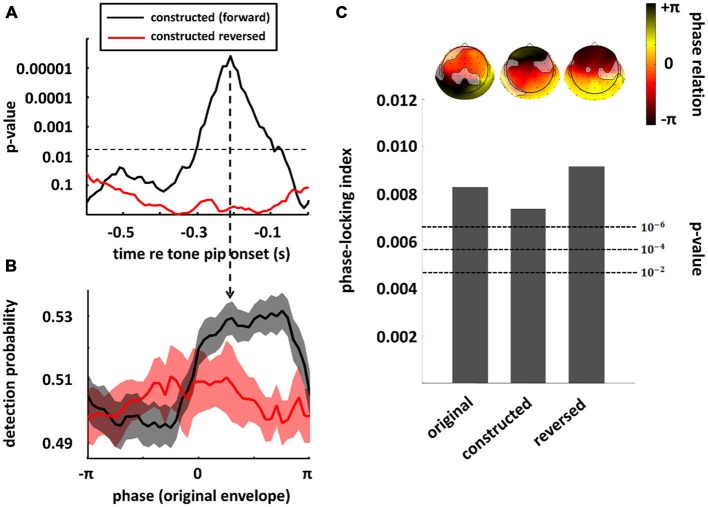
**Perception and neural oscillations entrain to high-level features of speech sound.** Speech/noise stimuli without systematic fluctuations in amplitude or spectral content were constructed, whose high-level features are conserved and reflected by the original speech envelope (cf. Figure 3). In a psychophysical study **(A,B)**, the detection of a tone pip was significantly modulated by those high-level features (black; in this plot, results obtained in the original experiment and a follow-up replication have been combined; both experiments are described in Zoefel and VanRullen, 2015b). The significance of this modulation is shown in **(A)** for different time lags relative to target onset, whereas the actual performance (at the time lag indicated by the vertical arrow in **(A)** is shown in panel **(B)**. This effect was abolished when the speech/noise sound was reversed (red), indicating an important role of linguistic features (i.e., intelligibility) for behavioral consequences of the entrainment. In **(A)**, the significance threshold is shown as a dashed line (corrected for multiple comparisons). In **(B)**, standard error of mean (SEM) is shown by contours around the lines. When the same stimuli (and their original version of everyday speech) were presented in an EEG experiment **(C)** significant phase-locking between original speech envelope and EEG signal could be observed in all conditions (original, speech/noise sound and reversed speech/noise sound), suggesting that high-level features can entrain the phase of EEG oscillations, and do so even if the speech is unintelligible (note that acoustic high-level features remain present in the speech/noise sound, even when it is reversed, as the listener can still differentiate speech and noise). Bars show the average phase-locking across EEG channels, whereas the actual phase differences between EEG signal and original speech envelope, separately for each channel, are shown as insets above the bars (channels without significant entrainment are shaded out). *P*-values of phase entrainment, obtained by permutation tests, are shown as dashed lines. Note that, in contrast to the degree of entrainment which is comparable in all three conditions, the entrained phase does differ between everyday speech sound (original condition) and speech/noise sound in which systematic fluctuations in low-level features have been removed (constructed and constructed reversed conditions). Modified with permission from Zoefel and VanRullen (2015b) **(A,B)** and Zoefel and VanRullen (2015a), copyright Elsevier **(C)**.

In the previous section, we have seen that high-level mechanisms of the brain, related to attention or prediction, clearly contribute to phase entrainment to speech sound. However, it should be noted that this contribution may just be *modulatory*: high-level mechanisms could merely *influence* a process, namely phase entrainment, that *itself* might rely on purely low-level processes. Indeed, speech sound consists of large fluctuations in low-level properties (i.e., stimulus amplitude and spectral content) that might evoke systematic fluctuations in neural activity already at the earliest level of auditory processing: the cochlea. These fluctuations in neural activity accompanying changes in the speech envelope would be indistinguishable from an active entrainment response. It is therefore necessary to construct stimuli without systematic fluctuations in those low-level properties in order to prove genuine high-level entrainment. In a recent publication (Zoefel and VanRullen, 2015b), we were able to construct such stimuli and we review the most important findings in this section, together with supporting results from other studies. Figure 3 shows the idea underlying stimulus construction in Zoefel and VanRullen (2015b). In everyday speech sound (Figure 3A), spectral energy (color-coded) clearly differs between different phases of the speech envelope. In the view of a single cochlear cell, this sound would periodically alternate between weak (e.g., at phase ± pi, which is the trough of the speech envelope) and strong excitation (e.g., at phase 0, which is the peak of the speech envelope). Consequently, at a larger scale, we would measure an oscillatory pattern of neural activity that strongly depends on envelope phase. This pattern, however, would only reflect the periodicity of the stimulation. Therefore, we constructed noise sound whose spectral energy was tailored to counterbalance spectral differences as a function of envelope phase of the original speech sound (for details of stimulus construction, see Zoefel and VanRullen, 2015b). This noise was mixed with the original speech and resulted in speech/noise sound that did, on average, not show those systematic differences in spectral content anymore (Figure 3B). Critically, as those stimuli remain intelligible, high-level features of speech (such as, but not restricted to, phonetic information) are still present and enable the listener to entrain to the speech sound that is now “hidden” inside the noise (note that the degree to which the speech is “hidden” in noise depends on the original envelope phase, with speech perceptually dominant at the original envelope peak, and noise perceptually dominant at the original envelope trough). We applied those stimuli in two studies: in the first (Zoefel and VanRullen, 2015b), a psychophysical study, we found that the detection of a short tone pip was significantly modulated (*p*-values shown in Figure 4A) by the remaining high-level features. Performance (Figure 4B) depended on the original envelope phase and thus differed between periods of dominant speech and noise. Note that speech and noise were spectrally matched; differences in performance could thus not be due to spectral differences between speech and noise, but rather due to the remaining high-level features that enable the listener to differentiate speech and noise. In the second study (Zoefel and VanRullen, 2015a), those stimuli were presented to listeners while their EEG was recorded. We found that EEG oscillations phase-lock to those high-level features of speech sound (Figure 4C), and the degree of entrainment (but not the phase relation between speech and EEG signal; see insets in Figure 4C) was similar to when the original everyday speech was presented. These results suggest an entrainment of neural oscillations as the mechanism underlying our perceptual findings.

It is not only interesting to investigate phase entrainment to speech stimuli *without* potentially entraining low-level features, but also to speech stimuli *only* containing the latter. This was done in a study by Ding et al. (2013) that might be seen as complementary to the other two described in this section. In their study, noise-vocoding (Green et al., 2002) was used in order to design stimuli where spectro-temporal fine structure (which can be considered as high-level features) was strongly reduced, but the speech envelope was essentially unchanged. Those stimuli were presented either in noise or in quiet, and MEG was recorded in parallel. Ding et al. (2013) showed that, indeed, the reduction of spectro-temporal fine structure in noise-vocoded speech results in a decline in phase entrainment as compared to that in response to natural speech sound. This result suggests that oscillations do not merely (and passively) follow the slow fluctuations in low-level features of speech (e.g., the speech envelope), as they are present in both natural and noise-vocoded speech. Instead, phase entrainment to speech sound involves an additional adjustment to rhythmic changes in spectro-temporal fine structure. It is important to mention that the effect was only observed in noise (and not in quiet), stressing the idea that separating speech and noise might be one of the main functions of phase entrainment to speech sound (see “Phase Entrainment as a Tool for Input Gating” and “High-Level Modulations of Phase Entrainment to Speech Sound”). Using similar stimuli as in Ding et al. (2013), Rimmele et al. (2015) both extended their findings and built a bridge to our section “High-Level Modulations of Phase Entrainment to Speech Sound”. In contrast to Ding et al. (2013), they presented natural and noise-vocoded speech concurrently and asked their subjects to attend one of them while ignoring the other. Interestingly, they were able to show that the enhanced “envelope tracking” for natural compared to noise-vocoded speech (as in Ding et al., 2013) is only present when the speech is attended. They interpret their results as evidence for a high-level mechanism (“linguistic processing”) that is only possible when speech is in the focus of the listener’s attention, and only when speech contains spectro-temporal fine structure (i.e., high-level features). Finally, no attentional modulation of phase entrainment was found for noise-vocoded speech which might be taken as evidence for a tracking of low-level features that does not depend on top-down processes (e.g., attention; see “High-Level Modulations of Phase Entrainment to Speech Sound”).

Taken together, the results reported in this section suggest that phase entrainment to speech sound is not only a reflection of fluctuations in low-level features of speech sound, but entails an adaption to phonetic information—and thus a genuine high-level process.

As briefly mentioned before, there is an ongoing debate which is directly related to the results presented in this section: as shown by Capilla et al. (2011), seemingly entrained oscillations can be explained by a superposition of evoked responses (see also Keitel et al., 2014). Transferring this result to speech sound, it has been argued specifically that a phase-reset of neural oscillations by (e.g.) “acoustic edges” of speech might be an important mechanism underlying phase entrainment (Doelling et al., 2014; Howard and Poeppel, 2010)—assuming that these “edges” occur regularly in speech, a periodic sequence of phase-resets might thus be sufficient to explain the observed “phase entrainment”. This paragraph provides arguments against phase entrainment reflecting a purely passive mechanism, reflecting merely sequences of neural phase-resets or evoked potentials; however, we do emphasize here that most studies likely measure a mixture of evoked and entrained neural responses. As already outlined above, the two studies described in the first paragraph of this section (Zoefel and VanRullen, 2015a,b) support the notion that phase entrainment is more than a steady-state response to rhythmic stimulation: it can be observed even when the presented speech sound does not contain systematic fluctuations in amplitude or spectral content. Indeed, there are more studies, using simpler, non-speech stimuli, that also support this conclusion. For instance, it has been found, for both vision (Spaak et al., 2014) and audition (Hickok et al., 2015), that behavioral performance fluctuates for several cycles after the offset of an entraining stimulus. A mere “following” of the stimulation would not produce these after-effects. Moreover, using entraining stimuli at threshold level, it has been shown that neural oscillations (as measured with EEG) entrain to the stimulation rate even when the stimulus is not perceived (e.g., in the case of several subsequent “misses”) and would therefore not evoke a strong neural response (Zoefel and Heil, 2013). Finally, in a clever experimental design, Herring et al. (2015) measured visual alpha oscillations (~8–12 Hz) after a single pulse of transcranial magnetic stimulation (TMS) that has previously been hypothesized to re-set (or entrain, in the case of multiple, rhythmic TMS pulses) endogenous oscillations (Thut et al., 2011). They then asked the question: how is the measured “alpha” modulated by attention? In the case of a simple evoked response (or “alpha-ringing”), the observed “alpha” would exhibit an increased amplitude when attention is allocated to the visual domain; however, in the case of endogenous alpha, visual attention would decrease the alpha amplitude, as described already by Adrian (1944). Indeed, the latter is what Herring et al. (2015) observed. To conclude, although the issue remains open, there are promising first results suggesting that phase entrainment—to speech or other stimuli, including brain stimulation—is more than steady-state responses evoked by the rhythmic stimulation—it entails high-level processes and an adjustment of endogenous neural oscillations.

## The Role of Intelligibility for Phase Entrainment to Speech Sound

Of course, the ultimate goal of every conversation is to transmit information, and without intelligibility, this goal cannot be achieved. Thus, it is all the more surprising that the role of intelligibility for phase entrainment to speech is currently strongly debated. This controversy is due to seemingly contradictory results that have been published. On the one hand, both Ahissar et al. (2001) and Luo and Poeppel (2007) found a correlation between phase entrainment (i.e., alignment of delta/theta oscillations and speech envelope) and speech intelligibility, a finding that has been confirmed by recent studies (Ding et al., 2013; Doelling et al., 2014; Park et al., 2015). On the other hand, phase entrainment is not a phenomenon that is unique to speech sound and can also be found in response to much simpler stimuli, such as pure tones (Lakatos et al., 2005, 2008; Stefanics et al., 2010; Besle et al., 2011; Gomez-Ramirez et al., 2011; Zoefel and Heil, 2013). Also, the manipulation of speech intelligibility might destroy acoustic (i.e., non-semantic) properties of the sound that the brain actually entrains to (such as acoustic “edges”; Doelling et al., 2014), leading to a decline in phase entrainment and speech intelligibility at the same time, but without any relation between the two (Peelle and Davis, 2012; Millman et al., 2015). Moreover, several studies showed phase entrainment of neural oscillations to unintelligible speech sound (Howard and Poeppel, 2010; Peelle et al., 2013; Millman et al., 2015) suggesting that phase entrainment does not necessarily depend on intelligibility. The whole picture gets even more complicated, as, although phase entrainment to speech sound is possible even when the speech is unintelligible, is seems to be enhanced by intelligible speech in some (but not all) studies (Gross et al., 2013; Peelle et al., 2013; Park et al., 2015) and attention seems to be important for this enhancement (Rimmele et al., 2015). Further evidence that the role of intelligibility for phase entrainment is not trivial was reported in two of the studies described in the previous section. In Zoefel and VanRullen (2015b), it was found that perceptual entrainment to high-level features of speech sound is disrupted when the speech/noise sound is reversed (Figures 4A,B; red line) and this result was interpreted as a critical role of intelligibility for perceptual phase entrainment. On the other hand, in Zoefel and VanRullen (2015a), using the same reversed speech/noise stimuli, the observed EEG phase entrainment was similar to that obtained in response to everyday speech and to (forward) speech/noise sound (Figure 4C), seemingly in contradiction to the behavioral results obtained in Zoefel and VanRullen (2015b).

How can we reconcile these studies, some of them clearly arguing against, and some for an important role of intelligibility for phase entrainment? Based on the current state of research, it is important to avoid overhasty conclusions and our interpretations have to remain speculative. Overall, phase entrainment seems to be a necessary, but not sufficient condition for speech comprehension. Speech intelligibility might not be possible without phase-locking, as we are not aware of any study reporting intelligible stimuli without oscillations (or perception) aligned to critical (low- and high-level) features of the speech sound. On the other hand, neural oscillations entrain to rhythmic structures (including reversed speech) even in the absence of intelligibility. It is clear that phase entrainment is a much more general phenomenon, and the brain might continuously scan its input for rhythmic patterns (indeed, popularity for auditory rhythms can be found in all cultures across the world and synchronization with rhythms—e.g., by clapping or dancing—is a general reaction to them). Once a rhythmic pattern has been detected, neural oscillations will align their phase to it (operating in the “rhythmic mode” described in Schroeder and Lakatos, 2009; see also Zoefel and Heil, 2013). Based on this notion, neural oscillations might always align to sound, as long as a rhythmic pattern can be detected (note that even the reversed speech/noise sound used in Zoefel and VanRullen, 2015a,b, contains a rhythmic pattern, as speech and noise can perceptually be differentiated). But what is the role of intelligibility? It is important to find a model that is at the same time parsimonious and can explain most results described in the literature. These findings are shortly summarized in the following:

1. Rhythmic non-speech stimuli, such as trains of pure tones, entrain neural oscillations (Lakatos et al., 2005; Besle et al., 2011; Gomez-Ramirez et al., 2011; Zoefel and Heil, 2013) and modulate behavior (Lakatos et al., 2008; Stefanics et al., 2010; Thut et al., 2012; Hickok et al., 2015).
2. Speech stimuli, both intelligible and unintelligible, entrain neural oscillations (Ahissar et al., 2001; Luo and Poeppel, 2007; Howard and Poeppel, 2010; Ding and Simon, 2012a; Ding et al., 2013; Peelle et al., 2013; Zion Golumbic et al., 2013b; Doelling et al., 2014; Millman et al., 2015; Park et al., 2015; Rimmele et al., 2015; Zoefel and VanRullen, 2015a).
3. The rhythm of speech only modulates behavior when speech is intelligible (Zoefel and VanRullen, 2015b).
4. Neural entrainment to intelligible speech might be increased when compared to unintelligible speech (Luo and Poeppel, 2007; Peelle et al., 2013; Doelling et al., 2014; Park et al., 2015; Rimmele et al., 2015). However, not all studies can confirm this result (Howard and Poeppel, 2010; Millman et al., 2015; Zoefel and VanRullen, 2015a).

**Figure 5:**
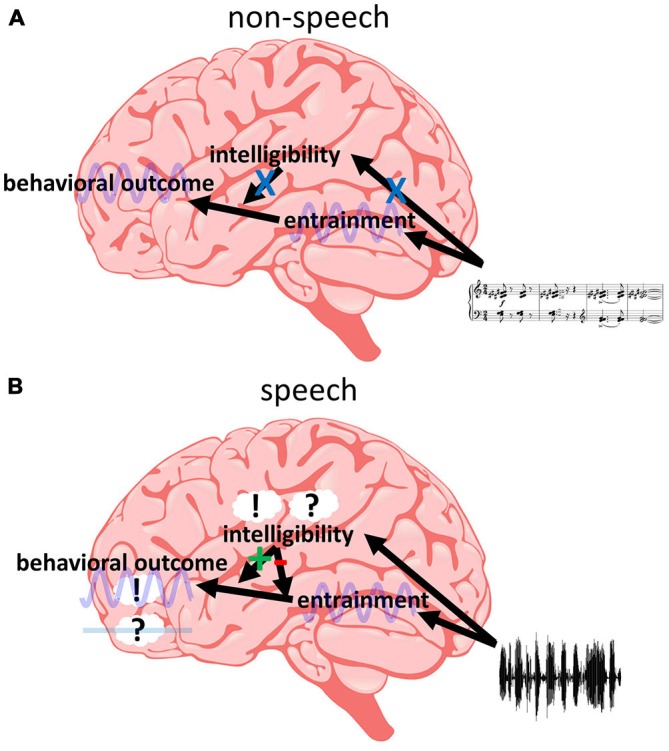
**Intelligibility at the interface between phase entrainment and behavior. (A)** Non-speech stimuli do not activate speech-specific (“intelligibility”) regions. Thus, entrainment in temporal regions can directly influence behavior—determined in frontal regions—in a periodic fashion, without an additional modulation by speech-specific regions. **(B)** Acoustic high-level features of speech activate speech-specific regions. This activation results in a modulation of the connectivity between temporal and frontal regions. If linguistic high-level features are present in the input (i.e., if the speech is intelligible), temporal and frontal regions are synchronized and entrainment in temporal regions can affect activity in frontal regions (and modulate behavior periodically, such as in **A**). If these features are not present (i.e., if the speech is unintelligible), temporal and frontal regions are desynchronized and entrainment in temporal regions cannot affect frontal regions and behavior. Thus, (only) if the input is recognized as speech, intelligibility can act as a “switch”, determining the influence of entrained oscillations on behavioral outcome.

One model that can potentially reconcile these findings is presented in Figure 5, and the different parts and implications of this model are discussed in the following. However, we acknowledge that it is only one out of possibly several candidate models to explain the data available in the literature. Nevertheless, in our view, this model is currently the most parsimonious explanation for existing findings and we therefore focus our review on it. The first implication of our model is that different regions in the brain are “responsible” for different processes: Phase entrainment might be found throughout the whole auditory system, but most studies emphasize primary auditory cortex (A1; Lakatos et al., 2005, 2013; O’Connell et al., 2014) or early temporal regions (Gomez-Ramirez et al., 2011; Ding and Simon, 2012b; Zion Golumbic et al., 2013b). An influence of intelligibility is commonly related to regions specifically processing speech sound (Binder et al., 2000; Scott et al., 2000; Hickok and Poeppel, 2007; DeWitt and Rauschecker, 2012; Poeppel et al., 2012; Mesgarani et al., 2014). Finally, frontal regions are a likely candidate for behavioral outcome (Krawczyk, 2002; Coutlee and Huettel, 2012; Rushworth et al., 2012; Romo and de Lafuente, 2013). In order to satisfy point (1), we assume that the entrainment in temporal regions can directly influence behavior as determined in frontal regions, as long as the entrainment is introduced by non-speech stimuli (Figure 5A). This results in a periodic modulation of performance as often described (Fiebelkorn et al., 2011; Vanrullen and Dubois, 2011; Landau and Fries, 2012; Thut et al., 2012; Song et al., 2014; Spaak et al., 2014; Zoefel and Sokoliuk, 2014; Hickok et al., 2015; note, however, that most studies report effects for the visual and not for the auditory system—it needs to be clarified whether this fact is biased by the number of studies investigating the visual system or whether there are genuine differences between the two systems). But not only non-speech stimuli can entrain temporal regions, the same is true for speech sound, irrespective of its intelligibility (point 2). However, speech intelligibility affects high-order auditory regions and they might directly influence the impact of temporal on frontal regions (Figure 5B). This notion is based on the increasing number of studies supporting the idea that the state of connectivity (or synchronization) between two (potentially distant) brain regions is crucial for perceptual outcome (Fries, 2005; Ruhnau et al., 2014; Weisz et al., 2014). Thus, speech intelligibility might modulate the state of connectivity between temporal and frontal regions. We hypothesize that speech-specific regions only become responsive if the input contains *acoustic* high-level (i.e., speech-specific) features of speech; otherwise these regions remain irrelevant and do not exhibit any modulatory effect on other regions or their connectivity. However, once the input is identified as speech (based on these acoustic features), *linguistic* features determine whether the modulatory effect is negative (desynchronizing temporal and frontal regions, resulting in no behavioral effect of the entrainment; in case of unintelligible speech) or positive (synchronizing temporal and frontal regions, resulting in a behavioral effect of the entrainment; in case of intelligible speech). This assumption satisfies point (3). In contrast to unintelligible speech, intelligible speech might result in an entrainment that also includes high-order (speech-specific) auditory regions. They might have to entrain to the speech sound in order to be able to synchronize temporal and frontal regions. That might be the reason that some studies show an increased entrainment for intelligible as compared to unintelligible speech whereas others do not (point 4). They might have captured the entrainment in those higher-level auditory regions—something which, due to the low spatial resolution in most EEG/MEG studies, is difficult to determine but could be resolved in future studies. More research is clearly needed: what are those behavioral variables that are differentially affected by intelligible and unintelligible speech? Where exactly are those brain regions hypothesized to be responsible for (or affected by) phase entrainment, for behavioral decisions and for the modulation of their relation by speech intelligibility? What are the mechanisms connecting these functional networks? Answering these questions has critical implications for our understanding of the brain’s processing of human speech and rhythmic input in general.

## Conclusion

Recently, phase entrainment has attracted researchers’ attention as a potential reflection of the brain’s mechanism to efficiently allocate attentional resources in time (for a recent review, see, e.g., Frey et al., 2015). Nevertheless, the periodicity of the stimulation itself complicates this interpretation, as the brain might simply follow the rhythm of its input. In this review, we presented an increasing amount of evidence that speaks against a merely passive role of neural oscillations for phase entrainment to speech sound. Instead, the brain might constantly predict the timing of relevant and irrelevant events of speech sound, including acoustic high-level features, and actively align neural oscillations so that they efficiently boost the current locus of attention in a noisy background. Linguistic high-level features, reflecting intelligibility, might play a modulatory, and speech-specific, role by determining the behavioral consequences of phase entrainment to speech sound.

## Conflict of Interest Statement

The authors declare that the research was conducted in the absence of any commercial or financial relationships that could be construed as a potential conflict of interest.
